# Comparison of vascular access outcomes in patients with end-stage renal disease attributed to systemic lupus erythematosus vs. other causes: a retrospective cohort study

**DOI:** 10.1186/s12882-016-0274-y

**Published:** 2016-07-07

**Authors:** Laura C. Plantinga, S. Sam Lim, Rachel E. Patzer, Stephen O. Pastan, Cristina Drenkard

**Affiliations:** Division of Renal Medicine, Department of Medicine, Emory University, 101 Woodruff Circle, 5105 Woodruff Memorial Building, Atlanta, GA 30322 USA; Division of Rheumatology, Department of Medicine, Emory University, Atlanta, GA USA; Division of Transplantation, Department of Surgery, Emory University, Atlanta, GA USA; Emory Transplant Center, Emory Healthcare, Emory University, Atlanta, GA USA

## Abstract

**Background:**

U.S. hemodialysis patients with systemic lupus erythematosus (SLE) and end-stage renal disease (ESRD) are less likely than other ESRD patients to have a permanent vascular access (fistula or graft) in place at the dialysis start. We examined whether vascular access outcomes after dialysis start differed for SLE vs. other ESRD patients.

**Methods:**

Among U.S. patients initiating hemodialysis in 2010 with only a catheter (*n* = 40,911; 384 with SLE) and using a permanent access on first dialysis (*n* = 13,073; 48 with SLE), we examined the association of SLE status with time to first placement of a permanent access (among catheter-only patients) and to loss of access patency (among patients using a permanent access on first dialysis), both censored 1 year after dialysis start, using multivariable Cox proportional hazards models.

**Results:**

Among catheter-only patients, 46.1 % vs. 54.5 % of those with SLE-ESRD vs. other ESRD had a permanent access placed within 1 year after dialysis start. However, with adjustment, there was no association of 1-year placement with SLE status [HR = 1.00 (95 % CI, 0.86-1.17)]. SLE-ESRD vs. other ESRD patients starting dialysis with a permanent access were less likely to experience a 1-year loss of patency (43.8 % vs. 55.0 %), but this association was not statistically significant after adjustment [HR = 0.88 (0.57-1.37)].

**Conclusion:**

These results suggest that SLE-ESRD patients starting dialysis with a catheter are not more likely to have a permanent access placed in the first year of dialysis, despite an observed lack of association of SLE status with subsequent loss of vascular access patency among those starting dialysis with a permanent access.

**Electronic supplementary material:**

The online version of this article (doi:10.1186/s12882-016-0274-y) contains supplementary material, which is available to authorized users.

## Background

End-stage renal disease (ESRD) patients who undergo hemodialysis with a permanent vascular access [arteriovenous fistula (AVF) or grafts (AVG)] rather than a temporary catheter generally have better outcomes [1–7] and lower associated healthcare costs [8]. Thus, national U.S. clinical guidelines promote the early placement of AVFs and AVG (at least 6 months and 3–6 weeks prior to anticipated dialysis start, respectively), such that a mature, functioning vascular access is in place on first dialysis [9]. Centers for Medicare & Medicaid Services (CMS) tracks early placement through the ESRD Medical Evidence form (CMS-2728), which collects data on access type at first dialysis for all patients starting ESRD treatment. CMS also promotes the placement of a permanent vascular access among hemodialysis patients through regionally implemented quality incentive programs such as Fistula First, Catheter Last [10].

Previously, we found that systemic lupus erythematosus (SLE) patients who developed ESRD were 40 % less likely to have a permanent vascular access in place at the start of dialysis than other ESRD patients, despite being more likely than other ESRD patients to have other indicators of advanced ESRD care planning, such as receipt of pre-ESRD nephrology care and earlier placement on the deceased donor kidney transplant waitlist [11, 12]. Further, O’Shaughnessy et al. [13] recently noted that ESRD patients with SLE were also less likely than ESRD patients with other types of glomerulonephritis to have an AVF in place at dialysis start. Here, using national claims data on ESRD patients, we examined whether subsequent placement of permanent vascular access differed for SLE-ESRD patients compared to other ESRD patients who started hemodialysis with only a catheter. Additionally, among U.S. patients who used a permanent access on their first hemodialysis treatment, we examined whether early loss of patency, as evidenced by procedures aimed at maintaining vascular access patency or placement of a new vascular access, differed for SLE-ESRD vs. other ESRD patients.

## Methods

### Study population and data sources

**Fig. 1:**
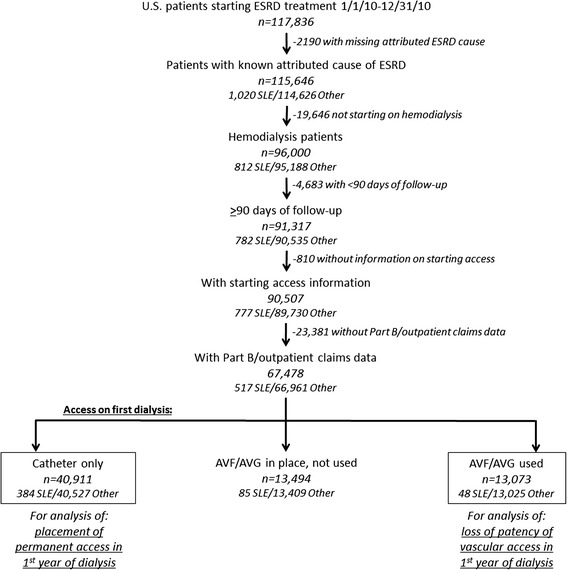
Selection of study population among U.S. 2010 incident dialysis patients, overall and by systemic lupus erythematosus-attributed and other end-stage renal disease. AVF, arteriovenous fistula; AVG, arteriovenous graft; ESRD, end-stage renal disease; SLE, systemic lupus erythematosus

For this retrospective cohort study, data from the CMS-2728 and Part A (inpatient) and Part B (outpatient) claims were obtained from the United States Renal Data System (USRDS) [14]. The study was approved by the Emory Institutional Review Board. Analyses were limited to patients starting dialysis in 2010, the most recent year available such that all patients had at least a year of potential follow-up. A total of 117,836 incident adult and pediatric ESRD patients were identified who initiated treatment from 1/1/10 to 12/31/10. Patients were excluded if they had a missing attributed cause of ESRD [*n* = 2,190 (1.9 %)]; did not start on hemodialysis [*n* = 19,646 (17.0 %); had fewer than 90 days of hemodialysis [*n* = 4,683 (4.9 %)], did not have information on starting vascular access [*n* = 810 (0.9 %)], or did not have evidence of Part B claims [*n* = 23,381 (25.8 %)], leaving 67,478 patients (517 with SLE-ESRD and 66,961 with other ESRD; Fig. 1). Patients without Part B claims were excluded to avoid differential ascertainment of outpatient vascular access events among those who opted out of Part B coverage.

### Study variables

#### SLE status

SLE-ESRD was defined as ESRD attributed to CMS-2728 ICD-9 code 710.0 (SLE). Other ESRD (the referent group) included all other non-missing ICD-9 codes listed on the CMS-2728.

#### Vascular access at dialysis start

Vascular access at dialysis start was determined from the CMS-2728. Vascular access at dialysis start was categorized as: having a catheter only [no permanent access (AVF or AVG) in place], having a permanent access in place but not used on first dialysis, and having a permanent access that was used on first dialysis.

#### Vascular access placement in first year of dialysis

Placement of a permanent access (AVF or AVG) was determined from Parts A and B claims data using Healthcare Common Procedure Coding System (HCPCS) codes (Additional file 1: Table S1). The date of placement was defined as the first date after start of dialysis in which an inpatient or outpatient code for placement was used.

#### Loss of vascular access patency in first year of dialysis

Loss of patency of a permanent access (AVF or AVG) was similarly determined from Part A and B claims data using HCPCS codes for revision procedures on the starting access or placement of new accesses (AVF, AVG, or catheter; Additional file 1: Table S1). The date of loss of patency was defined as the first date after start of dialysis on which an inpatient or outpatient code for a revision or placement was used.

#### Other variables

Incident age, sex, and treatment modalities were obtained from the USRDS standard analytic files. Race/ethnicity, primary insurance at start of ESRD, smoking status, body mass index (BMI), and presence of comorbid conditions were all obtained from the CMS-2728. Race/ethnicity was categorized as white, black, Hispanic, or other. Primary insurance at start of ESRD was categorized as private, Medicaid, none, or other (including Medicare, which is rare in SLE patients).

### Statistical analysis

Patient characteristics were summarized overall and by attributed cause and vascular access at dialysis start. For analysis of placement of permanent access within the first year of dialysis, 40,911 patients with a catheter only at start of dialysis (Fig. 1) were included; for analysis of loss of patency of permanent access within the first year of dialysis, 13,073 patients with an AVF or AVG that was used on first dialysis (Fig. 1) were included.. The outcomes were time from dialysis start to first placement of AVF or AVG and time from start to first revision of the starting access or placement of a new access (Additional file 1: Table S1), with censoring for transplant, switch to peritoneal dialysis, death, or end of follow-up (1 year after dialysis start). Separate analyses for AVF and AVG placement were censored for placement of the other type of permanent access. Additional sensitivity analyses (*i*) excluded children (age <18 years), to account for greater likelihood of pediatric ESRD onset in SLE- vs. non-SLE-ESRD; (*ii*) included only those with Medicare at ESRD start, to account for differential capture of events in the first 90 days of dialysis; (*iii*) included a comparison group of other glomerulonephritis patients [13], to examine whether SLE patients less likely than these potentially more comparable patients to have a permanent access placed; (*iv*) used 2:1 matching on age, sex, and race rather than statistical adjustment; and (*v*) included all events prior to day 90 on day 91 and (*vi*) excluded all events prior to day 90 on the estimates obtained in primary analyses, also to account for potential differential capture of events prior to day 91, when Medicare ESRD coverage for those patients <65 and not disabled begins. This scenario may be more likely in SLE, given patients’ younger age at ESRD start. For both placement and loss of patency analyses, we generated Kaplan-Meier curves by SLE-ESRD vs. other ESRD and estimated hazard ratios (HRs) and CIs using crude and multivariable Cox proportional hazards models, with adjustment for age, sex, race/ethnicity, BMI, comorbid conditions, and smoking and using complete case analysis. Outcomes were not examined among the 13,494 remaining patients (Fig. 1) who had an access in place that was not used on first dialysis because the date of first use of the access cannot be determined from claims data. Stata v. 14 (StataCorp, College Station, TX) was used for all analyses and we adhered to STROBE guidelines for reporting.

## Results

### Characteristics of the study population by SLE status and vascular access at dialysis start

Patients with ESRD attributed to SLE were younger (mean age, 40.3 vs. 64.3 years), more likely to be female (79.3 % vs. 43.1 %) and black (54.9 % vs. 29.8 %), and more likely to have Medicaid coverage at dialysis start (39.0 % vs. 26.9 %), compared to patients with other attributed causes of ESRD (*P* < 0.001 for all; Table 1). SLE-ESRD patients were less likely than other ESRD patients to have comorbid conditions and to smoke (Table 1). While low overall, transplantation (2.5 % vs. 1.3 %, *P* = 0.01) and renal recovery (2.5 % vs. 1.6 %; not statistically significant) within a year of dialysis start were both more likely in SLE-ESRD than other ESRD patients (Table 1).

**Table 1:** Characteristics of 2010 incident U.S. hemodialysis patients, by vascular access at dialysis start and systemic lupus erythematosus status

Characteristic	Overall	Vascular Access on First Dialysis:							
		Any		Catheter only		AVF/AVG in place		AVF/AVG used	
		Attributed Cause of ESRD:							
		SLE	Other	SLE	Other	SLE	Other	SLE	Other
N (%)	67,748 (100 %)	517 (0.8 %)	66,961 (99.2 %)	384 (0.9 %)	40,527 (99.1 %)	85 (0.6 %)	13,409 (99.4 %)	48 (0.4 %)	13,025 (99.6 %)
Demographic									
Mean (SD) age, years	64.1 (14.9)	40.3 (15.3)^a^	64.3 (14.8)^a^	39.0 (15.0)^a^	64.1 (15.3)^a^	41.7 (15.8)^a^	63.8 (14.3)^a^	48.6 (14.2)^a^	65.4 (13.6)^a^
Female, n (%)	29,276 (43.4 %)	410 (79.3 %)^a^	28,866 (43.1 %)^a^	315 (82.0 %)^a^	17,951 (44.3 %)^a^	64 (75.3 %)^a^	5,675 (42.3 %)^a^	31 (64.6 %)^b^	7,785 (59.8 %)^b^
Race/ethnicity, %									
Non-Hispanic white	34,731 (51.5 %)	129 (25.0 %)^a^	34,602 (51.7 %)^a^	89 (23.2 %)^a^	20,813 (51.4 %) ^a^	22 (25.9 %)^a^	6,644 (49.6 %)^a^	18 (37.5 %)	7,145 (54.9 %)
Black	20,221 (30.0 %)	284 (54.9 %)^a^	19,937 (29.8 %)^a^	221 (57.6 %)^a^	11,984 (29.6 %)^a^	42 (49.4 %)^a^	4,108 (30.6 %)^a^	21 (43.8 %)	3,845 (29.5 %)
Hispanic white	8,722 (12.9 %)	72 (13.9 %)^a^	8,650 (12.9 %)^a^	56 (14.6 %)^a^	5,526 (13.6 %)^a^	10 (11.8 %)^a^	1,829 (13.6 %)^a^	—^c^	1,295 (9.9 %)
Other	3,804 (5.6 %)	32 (6.2 %)^a^	3,772 (5.6 %)^a^	18 (4.7 %)^a^	2,204 (5.4 %)^a^	11 (12.9 %)^a^	828 (6.2 %)^a^	—^c^	740 (5.7 %)
Insurance at dialysis start, %									
Private	16,546 (24.5 %)	137 (26.5 %)^a^	16,409 (24.5 %)^a^	93 (24.2 %)^a^	9,441 (23.3 %)^a^	25 (29.4 %)^b^	3,387 (25.3 %)^b^	19 (39.6 %)	3,264 (25.1 %)
Medicare/other	27,159 (40.3 %)	104 (20.1 %)^a^	27,055 (40.4 %)^a^	67 (17.5 %)^a^	16,037 (39.6 %)^a^	21 (24.7 %)^b^	5,315 (39.6 %)^b^	16 (33.3 %)	3,581 (27.5 %)
Medicaid	18,183 (27.0 %)	201 (38.9 %)^a^	17.982 (26.9 %)^a^	160 (41.7 %)^a^	10,897 (26.9 %)^a^	32 (37.7 %)^b^	3,821 (28.5 %)^b^	—^c^	5,703 (43.8 %)
None	5,590 (8.3 %)	75 (14.5 %)^a^	5,515 (8.2 %)^a^	64 (16.7 %)^a^	4,152 (10.3 %)^a^	—^c^	886 (6.6 %)^b^	—^c^	477 (3.7 %)
Clinical									
Pre-ESRD nephrology care, %	38,937 (65.7 %)	307 (67.0 %)	38,630 (65.7 %)	200 (60.2 %)^b^	18,042 (52.4 %)^b^	64 (81.0 %)	9,034 (74.4 %)	43 (91.5 %)	11,554 (94.3 %)
Mean (SD) BMI, kg/m^2^	29.5 (7.9)	27.3 (7.5)^a^	29.5 (7.9)^a^	27.2 (7.5)^a^	29.3 (8.0)^a^	27.7 (6.9)^a^	29.9 (7.9)^a^	27.3 (8.3)^b^	29.8 (7.7)^b^
Congestive heart failure, %	22,893 (33.9 %)	85 (16.4 %)^a^	22,808 (34.1 %)^a^	59 (15.4 %)^a^	14,292 (35.3 %)^a^	18 (21.2 %)^b^	4,819 (35.9 %)^b^	—^c^	3,697 (28.4 %)
Diabetes, %	38,918 (57.7 %)	62 (12.0 %)^a^	38,856 (58.0 %)^a^	44 (11.5 %)^a^	22,940 (56.6 %)^a^	12 (14.1 %)^a^	8,401 (62.7 %)^a^	—^c^	7,515 (57.7 %)^a^
Peripheral vascular disease, %	9,569 (14.2 %)	21 (4.1 %)^a^	9,548 (14.3 %)^a^	16 (4.2 %)^a^	5,494 (13.6 %)^a^	—^c^	2,267 (16.9 %)^b^	—^c^	1,787 (13.7 %)^b^
Smoking, %	4,369 (6.5 %)	26 (5.0 %)	4,343 (6.5 %)	13 (3.4 %)^b^	2,645 (6.5 %)^b^	10 (11.8 %)	889 (6.6 %)	—^c^	810 (6.2 %)
Transplanted within 1 year, %	853 (1.3 %)	13 (2.5 %)^b^	840 (1.3 %)^b^	—^c^	429 (1.1 %)^b^	—^c^	160 (1.2 %)	—^c^	252 (1.9 %)
Recovered renal function within 1 year, %	1048 (1.6 %)	13 (2.5 %)	1035 (1.6 %)	11 (2.9 %)	909 (2.2 %)	—^c^	85 (0.6 %)	—^c^	41 (0.3 %)

*AVF* arteriovenous fistula, *AVG* arteriovenous graft, *ESRD* end-stage renal disease, *SLE* systemic lupus eythematosus

^a^ *P* <0.001 and ^b^ *P* < 0.05 for SLE vs. other ESRD, by *t* or chi-square/Fisher’s exact test, as appropriate

^c^Not reportable due to cell size < 10

Most SLE-ESRD patients (74.3 %) started dialysis with only a catheter, while 9.3 % started dialysis using an AVF or AVG; in contrast, 60.5 % and 19.5 % of other ESRD patients started dialysis with a catheter only or using an AVF/AVG, respectively (*P* < 0.001). Within subgroups defined by starting vascular access, patterns by SLE status of patient characteristics were generally similar to the patterns in the overall dialysis patients (Table 1). For those using an AVF/AVG on first dialysis, differences by SLE status by female sex and black race were slightly attenuated, relative to the overall population (Table 1). SLE patients starting dialysis with an AVF/AVG were older (mean age, 48.6 years) and more likely to have private insurance than other SLE-ESRD patients (Table 1).

### Association of SLE status with vascular access outcomes

#### Vascular access placement within 1 year

**Fig. 2:**
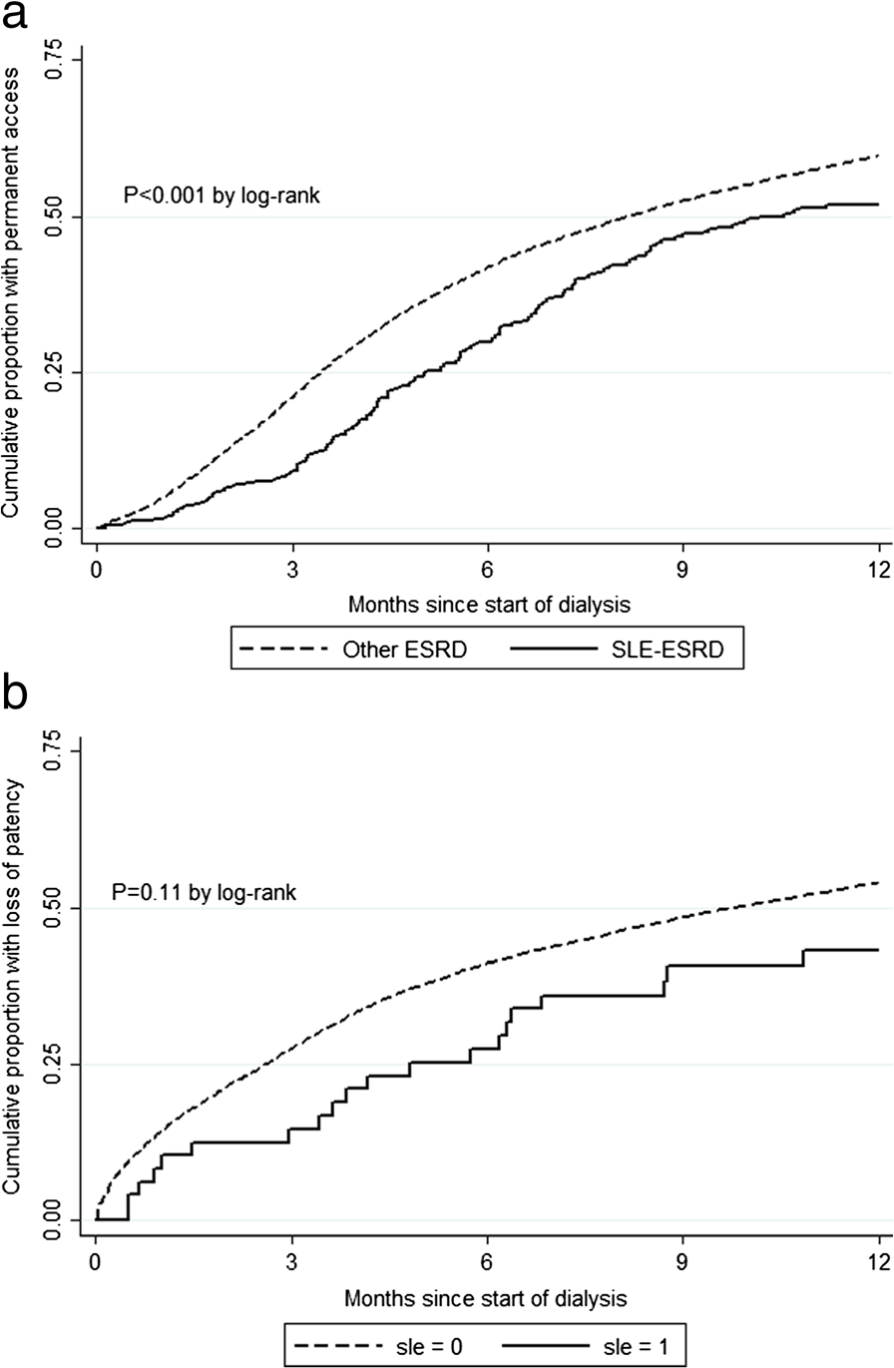
Time to access placement **a** and loss of access patency **b** in the first year of dialysis, among U.S. incident hemodialysis patients with systemic lupus erythematosus-attributed vs. other end-stage renal disease in 2010. **a**, among patients who started with only a catheter; **b**, among patients who used an arteriovenous fistula or graft on first dialysis. ESRD, end-stage renal disease; SLE, systemic lupus erythematosus

Among hemodialysis patients without a permanent access at dialysis start, 46.1 % of SLE-ESRD patients and 54.5 % of other ESRD patients had a permanent access placed within 1 year of dialysis start (Table 2). Crude rates of AVF/AVG placement over the first year of dialysis were 26 % lower among patients with SLE-ESRD who did not have a permanent access in place at dialysis start, relative to their counterparts with other ESRD (Fig. 2a; Table 2). However, adjustment for demographics and clinical characteristics rendered this association null (Table 2), and this attenuation was largely driven by adjustment for differences in ages between SLE-ESRD and other ESRD patients [age-adjusted HR: 0.96 (95 % CI, 0.83-1.12)]. Similar patterns were seen for AVF and AVG placement individually, although crude results were not statistically significant for AVG placement alone (Table 2). Sensitivity analyses showed results similar to those obtained in the primary analysis (Table 3).

**Table 2:** Association of placement of permanent vascular access with attribution of end-stage renal disease to systemic lupus erythematosus vs. other causes, among U.S. incident hemodialysis patients who started dialysis in 2010 with only a catheter in place

Model	No. (%) with permanent access placed within 1 year	Hazard ratio (95 % CI) for placement of permanent vascular access, SLE-attributed vs. other ESRD		
		Unadjusted	Adjusted for demographics	Adjusted for demographics + clinical
All events				
SLE-attributed ESRD	177 (46.1 %)	0.74 (0.64-0.86)	0.94 (0.81-1.09)	1.00 (0.86-1.17)
Other ESRD	22,076 (54.5 %)	1.00 (ref.)	1.00 (ref.)	1.00 (ref.)
*P*	*0.001*	*<0.001*	*0.42*	*>0.9*
All AVF placements^a^				
SLE-attributed ESRD	137 (35.7 %)	0.73 (0.62-0.87)	0.93 (0.79-1.11)	1.00 (0.84-1.18)
Other ESRD	17,300 (42.7 %)	1.00 (ref.)	1.00 (ref.)	1.00 (ref.)
*P*	*0.006*	*<0.001*	*0.43*	*>0.9*
All AVG placements^a^				
SLE-attributed ESRD	40 (10.4 %)	0.77 (0.56-1.05)	1.00 (0.73-1.38)	1.06 (0.77-1.47)
Other ESRD	4,776 (11.8 %)	1.00 (ref.)	1.00 (ref.)	1.00 (ref.)
*P*	*0.41*	*0.10*	*>0.9*	*0.71*

*AVF* arteriovenous fistula, *AVG* arteriovenous graft, *ESRD* end-stage renal disease, *SLE* systemic lupus eythematosus. Demographics: age (continuous), sex, race; clinical: body mass index (continuous), smoking, congestive heart failure, diabetes, and peripheral vascular disease

^a^Time-to-event analyses censored for placement of other type of access

**Table 3:** Association of placement of permanent vascular access with attribution of end-stage renal disease to systemic lupus erythematosus vs. other causes, among U.S. incident hemodialysis patients who started dialysis in 2010 with only a catheter in place: sensitivity analyses

Model	No. (%) with permanent access placed within 1 year	Hazard ratio (95 % CI) for placement of permanent vascular access, SLE-attributed vs. other ESRD		
		Unadjusted	Adjusted for demographics	Adjusted for demographics + clinical
Primary analysis				
SLE-attributed ESRD	177 (46.1 %)	0.74 (0.64-0.86)	0.94 (0.81-1.09)	1.00 (0.86-1.17)
Other ESRD	22,076 (54.5 %)	1.00 (ref.)	1.00 (ref.)	1.00 (ref.)
*P*	*0.001*	*<0.001*	*0.42*	*>0.9*
Excluding patients <18 years old^a^				
SLE-attributed ESRD	171 (46.2 %)	0.74 (0.64-0.86)	0.92 (0.79-1.08)	0.98 (0.84-1.15)
Other ESRD	22,053 (54.6 %)	1.00 (ref.)	1.00 (ref.)	1.00 (ref.)
*P*	*0.001*	*<0.001*	*0.31*	*0.82*
Only among patients with Medicare at ESRD start^b^				
SLE-attributed ESRD	67 (58.3 %)	0.77 (0.60-0.99)	0.83 (0.65-1.07)	0.83 (0.65-1.07)
Other ESRD	15,635 (64.0 %)	1.00 (ref.)	1.00 (ref.)	1.00 (ref.)
*P*	*0.20*	*0.04*	*0.15*	*0.15*
SLE and other GN compared to other ESRD^c^				
SLE-attributed ESRD	177 (46.1 %)	0.73 (0.63-0.85)	0.93 (0.80-1.08)	0.97 (0.83-1.14)
Other GN-attributed ESRD	1,219 (48.6 %)	0.83 (0.78-0.88)	0.91 (0.86-0.96)	0.94 (0.89-1.00)
Other ESRD	20,857 (54.9 %)	1.00 (ref.)	1.00 (ref.)	1.00 (ref.)
*P*	*<0.001*	*<0.001/<0.001*	*0.35/<0.001*	*0.74/0.06*
Matched analysis^d^				
SLE-attributed ESRD	177 (46.1 %)	0.85 (0.71-1.02)	0.88 (0.73-1.06)	0.93 (0.77-1.13)
Other ESRD	350 (51.7 %)	1.00 (ref.)	1.00 (ref.)	1.00 (ref.)
*P*	*0.08*	*0.09*	*0.17*	*0.48*
Events prior to 90 days included on day 91				
SLE-attributed ESRD	177 (46.1 %)	0.76 (0.65-0.88)	0.94 (0.81-1.10)	1.00 (0.86-1.17)
Other ESRD	22,076 (54.5 %)	1.00 (ref.)	1.00 (ref.)	1.00 (ref.)
*P*	*0.001*	*<0.001*	*0.46*	*>0.9*
Events prior to 90 days excluded^e^				
SLE-attributed ESRD	142 (40.7 %)	0.93 (0.79-1.10)	1.05 (0.89-1.25)	1.07 (0.91-1.27)
Other ESRD	13,653 (42.6 %)	1.00 (ref.)	1.00 (ref.)	1.00 (ref.)
*P*	*0.48*	*0.41*	*0.56*	*0.42*

*AVF* arteriovenous fistula, *AVG* arteriovenous graft, *ESRD* end-stage renal disease, *GN* glomerulonephritis, *SLE* systemic lupus eythematosus. Demographics: age (continuous), sex, race; clinical: body mass index (continuous), smoking, congestive heart failure, diabetes, and peripheral vascular disease

^a^Excluding *n* = 162 patients (14 SLE, 148 other) who were <18 years old

^b^Excluding *n* = 16,374 patients (269 SLE, 16,105 other) without Medicare at ESRD start

^c^Other GN included 2,511 patients with CMS-2728 ICD-9 codes for attributed cause of glomerulonephritis (582.9, 582.1, 583.1, 583.21, 583.22, 583.81, 583.82, 583.4, 580.0, and 582.0) or secondary glomerulonephritis/vasculitis (excluding SLE-ESRD; 287.0, 710.1, 283.11, 446.0, 446.4, 583.92, 446.20, 446.21, and 583.91)

^d^Among *n* = 1061 patients, including 384 SLE patients and 677 matched non-SLE patients, using 2:1 matching on age group (<30, 30–49, and >50 years), sex (female and male), and race (black and not black). Models stratified on matching variables to account for matching

^e^Excluding *n* = 8,491 patients (35 SLE, 8,456 other) with follow-up time <90 days

#### Loss of vascular access patency in first year of dialysis

SLE-ESRD patients using a permanent access (AVF/AVG) on first dialysis were less likely than their non-SLE counterparts to experience a loss of patency of that access within the first year (43.8 % vs. 55.0 %, *P* = 0.12; Table 4). SLE-ESRD patients had 30 % lower crude rates of access revision than other ESRD patients within the first year of dialysis, but the association was not statistically significant (Fig. 2b; Table 4). Adjustment for potential confounders attenuated the association. Results among those starting with an AVF were similar to the overall results, whereas results among those starting with an AVG showed a greater magnitude of effect (46 % lower rates of access revisions among SLE-ESRD vs. other ESRD patients) but without statistical significance, likely due to low numbers of individuals and events in the SLE-ESRD subgroup (Table 4). Results from sensitivity analyses excluding children were similar to results from the primary analysis (Table 5).

**Table 4:** Association of loss of vascular access patency^a^ with attribution of ESRD to SLE vs. other causes, among 2010 U.S. incident hemodialysis patients who started dialysis with a permanent vascular access

Model	No. (%) with access revision within 1 year	Hazard ratio (95 % CI) for loss of patency, SLE-attributed vs. other ESRD		
		Unadjusted	Adjusted for demographics	Adjusted for demographics + clinical
All patients				
SLE-attributed ESRD	21 (43.8 %)	0.70 (0.45-1.09)	0.81 (0.52-1.26)	0.88 (0.57-1.37)
Other ESRD	7,169 (55.0 %)	1.00 (ref.)	1.00 (ref.)	1.00 (ref.)
*P*	*0.12*	*0.11*	*0.35*	*0.58*
Among patients with AVF^b^				
SLE-attributed ESRD	19 (44.2 %)	0.74 (0.46-1.17)	0.86 (0.54-1.37)	0.94 (0.59-1.50)
Other ESRD	5,735 (52.9 %)	1.00 (ref.)	1.00 (ref.)	1.00 (ref.)
*P*	*0.26*	*0.20*	*0.53*	*0.80*
Among patients with AVG^b^				
SLE-attributed ESRD	—^c^	0.54 (0.13-2.15)	0.57 (0.14-2.30)	0.61 (0.15-2.47)
Other ESRD	1,434 (65.8 %)	1.00 (ref.)	1.00 (ref.)	1.00 (ref.)
*P*	*0.35*	*0.38*	*0.43*	*0.49*

*AVF* arteriovenous fistula, *AVG* arteriovenous graft, *ESRD* end-stage renal disease, *SLE* systemic lupus eythematosus. Demographics: age (continuous), sex, race; clinical: body mass index (continuous), smoking, congestive heart failure, diabetes, and peripheral vascular disease

^a^Defined as a revision or placement of a new AVF, AVG, or catheter (see Table 1)

^b^ *N* = 10,890 and 2,183 for AVF and AVG, respectively

^c^Not reportable due to cell size < 10

**Table 5:** Association of loss of vascular access patency^a^ with attribution of ESRD to SLE vs. other causes, among 2010 U.S. incident hemodialysis patients who started dialysis with a permanent vascular access: sensitivity analyses

Model	No. (%) with access revision within 1 year	Hazard ratio (95 % CI) for loss of patency, SLE-attributed vs. other ESRD		
		Unadjusted	Adjusted for demographics	Adjusted for demographics + clinical
Primary analysis				
SLE-attributed ESRD	21 (43.8 %)	0.70 (0.45-1.09)	0.81 (0.52-1.26)	0.88 (0.57-1.37)
Other ESRD	7,169 (55.0 %)	1.00 (ref.)	1.00 (ref.)	1.00 (ref.)
*P*	*0.12*	*0.11*	*0.35*	*0.58*
Excluding patients <18 years old^b^				
SLE-attributed ESRD	21 (43.8 %)	0.70 (0.45-1.08)	0.81 (0.52-1.26)	0.88 (0.57-1.37)
Other ESRD	7,165 (55.1 %)	1.00 (ref.)	1.00 (ref.)	1.00 (ref.)
*P*	*0.12*	*0.11*	*0.35*	*0.57*
Only among patients with Medicare at ESRD start^c^				
SLE-attributed ESRD	---^d^	0.91 (0.55-1.52)	0.86 (0.52-1.42)	0.92 (0.55-1.53)
Other ESRD	1125 (13.0 %)	1.00 (ref.)	1.00 (ref.)	1.00 (ref.)
*P*	*0.76*	*0.73*	*0.55*	*0.75*
SLE and other GN compared to other ESRD^e^				
SLE-attributed ESRD	---^d^	0.69 (0.44-1.07)	0.80 (0.52-1.24)	0.87 (0.56-1.35)
Other GN-attributed ESRD	60 (6.9 %)	0.79 (0.71-0.88)	0.87 (0.78-0.96)	0.91 (0.82-1.02)
Other ESRD	1433 (11.8 %)	1.00 (ref.)	1.00 (ref.)	1.00 (ref.)
*P*	*<0.001*	*0.10/<0.001*	*0.32/0.008*	*0.54/0.09*
Matched analysis^f^				
SLE-attributed ESRD	21 (43.8 %)	0.71 (0.42-1.19)	0.70 (0.42-1.15)	0.81 (0.47-1.40)
Other ESRD	89 (58.2 %)	1.00 (ref.)	1.00 (ref.)	1.00 (ref.)
*P*	*0.08*	*0.19*	*0.16*	*0.46*
Events prior to 90 days included on day 91				
SLE-attributed ESRD	21 (43.8 %)	0.73 (0.47-1.13)	0.81 (0.52-1.27)	0.88 (0.57-1.37)
Other ESRD	7,169 (55.0 %)	1.00 (ref.)	1.00 (ref.)	1.00 (ref.)
*P*	*0.12*	*0.16*	*0.36*	*0.57*
Events prior to 90 days excluded^g^				
SLE-attributed ESRD	13 (32.5 %)	0.85 (0.49-1.46)	0.76 (0.44-1.31)	0.84 (0.48-1.45)
Other ESRD	3,377 (36.6 %)	1.00 (ref.)	1.00 (ref.)	1.00 (ref.)
*P*	*0.59*	*0.56*	*0.32*	*0.52*

*AVF* arteriovenous fistula, *AVG* arteriovenous graft, *ESRD* end-stage renal disease, *GN* glomerulonephritis, *SLE* systemic lupus eythematosus. Demographics: age (continuous), sex, race; clinical: body mass index (continuous), smoking, congestive heart failure, diabetes, and peripheral vascular disease

^a^Defined as a revision or placement of a new AVF, AVG, or catheter (see Table 1)

^b^Excluding *n* = 14 patients (4 SLE, 10 other) who were <18 years old

^c^Excluding *n* = 4,375 patients (23 SLE, 4,352 other) without Medicare at ESRD start

^d^Not reportable due to cell size < 10

^e^Other GN included 864 patients with CMS-2728 ICD-9 codes for attributed cause of glomerulonephritis (582.9, 582.1, 583.1, 583.21, 583.22, 583.81, 583.82, 583.4, 580.0, and 582.0) or secondary glomerulonephritis/vasculitis (excluding SLE-ESRD; 287.0, 710.1, 283.11, 446.0, 446.4, 583.92, 446.20, 446.21, and 583.91)

^f^Among *n* = 201 patients, including 48 SLE patients and 153 matched non-SLE patients, using 2:1 matching on age group (<30, 30–49, and >50 years), sex (female and male), and race (black and not black). Models stratified on matching variable to account for matching

^g^Excluding *n* = 3,801 patients (35 SLE, 8,456 other) with follow-up time <90 days

## Discussion

We found that, among U.S. hemodialysis patients starting treatment in 2010 without a permanent access in place, those with SLE-ESRD were equally as likely as other ESRD patients to have an AVF or AVG placed in the first year of dialysis after adjustment for potential confounders, particularly age. These results, which were robust to sensitivity analyses, suggest that the differential prevalence of permanent access in place at the start of hemodialysis seen in prior studies of both adults (24 % vs. 36 % for SLE vs. other ESRD patients) [11–13] and children (23 % vs. 43 %) [15, 16] is not the result of SLE-ESRD patients being more likely than other ESRD patients to have their permanent access placed after, rather than before, hemodialysis start. Further, it suggests that the substantial gap in permanent access placement by SLE status at ESRD start does not narrow, even after a year on treatment, which may subject SLE-ESRD patients receiving hemodialysis to greater risk of morbidity and mortality [1–8] than their non-SLE counterparts.

One reason providers and patients might delay placement of permanent access in a patient approaching ESRD treatment that the patient is expected to receive a kidney transplant shortly after hemodialysis treatment begins. This scenario may be more common in SLE-ESRD patients, who tend to be younger and, potentially, better transplant candidates, as suggested by their relatively greater rates of placement on the deceased donor waitlist and transplantation [12, 17]. Here, we found that, while early transplantation was nearly twice as common among the SLE-ESRD vs. other ESRD patients with only a catheter for access at the start of hemodialysis, the absolute likelihood of transplantation within the first year of hemodialysis among these SLE-ESRD patients was only 2.5 %, suggesting that expected early transplantation is not a justification for lack of permanent access placement at or before hemodialysis start. Similarly, providers may be more likely to anticipate renal recovery in SLE-ESRD [18] and avoid vascular surgery referral, but we found that, while SLE patients who started dialysis with a catheter only were more likely to recover renal function within a year than their non-SLE counterparts, fewer than 3 % of these SLE patients recovered renal function. Thus, anticipated recovery is also not likely to play a large role in the continuing gap in permanent vascular access placement by SLE status among hemodialysis patients after the first year of hemodialysis treatment. The predominance of female sex among SLE-ESRD patients might be another reason for the continued disparity in vascular access placement by SLE status over the first year of hemodialysis, due to potentially greater difficulty placing AVFs [19] and greater prevalence of body image issues associated with permanent vascular access [20] among females. However, adjustment for sex did not change our results. Differential provider referrals for vascular access surgery by SLE status could be also affected by greater likelihood of intravenous treatment history and/or hypercoagulability in SLE patients, particularly those who may have anti-phospholipid syndrome [21]; data were not available to examine these potential factors.

We also found that, among hemodialysis patients who started treatment with a permanent vascular access, loss of patency of the vascular access was actually less common among SLE-ESRD than other ESRD patients. However, this association was not statistically significant, even before adjustment. These results suggest that any provider perceptions about increased risk of vascular access problems for SLE-ESRD patients vs. other ESRD patients on hemodialysis due to clinical features of SLE [21], which could lead to fewer referrals to vascular access surgery, could be overestimated or even unfounded. However, these results should be interpreted with caution due to the potential for confounding by indication. In other words, it is possible that SLE-ESRD patients who started hemodialysis with a permanent vascular access were referred for vascular access surgery because they were at lesser risk for loss of vascular access patency than other SLE-ESRD patients, due to clinical features of their SLE not captured in these administrative data. Thus, we can only conclude that, among those ESRD patients who are observed to have a permanent access placed prior to the start of hemodialysis, SLE status is likely not associated with greater risk of loss of patency.

We found that AVFs were placed more often than AVGs in both SLE-ESRD and other ESRD patients in the first year of dialysis, which is in line with national clinical practice guidelines [9] and quality improvement programs such as the Fistula First, Catheter Last initiative [10]. However, the null association of permanent vascular access placement with SLE status did not differ by type of permanent access placed. We also found that loss of vascular access patency was more common among patients starting hemodialysis with AVGs vs. AVFs, which is consistent with prior evidence that access outcomes are poorer in AVGs than AVFs [1, 22, 23]. Results were suggestive that SLE-ESRD patients with AVGs were less likely to experience loss of patency than other ESRD patients with AVGs, whereas there was no association of SLE status with revisions among patients with AVFs. However, differences by SLE status were not statistically significant, likely due to lack of power in subgroup analyses.

This study has several important strengths, including the capture of all U.S. patients treated for ESRD, limited loss to follow-up due to universal coverage of ESRD services by CMS, completion of the CMS-2728 for all treated patients, and availability of claims data for all inpatient and outpatient services after start of dialysis. However, there are limitations, in addition to those noted above, which deserve mention. There is the potential for selection bias in the exclusion of a large number of patients without Medicare Part B coverage, and SLE-ESRD patients were more likely than other ESRD patients (33.5 % vs. 25.4 %) to be excluded due to lack of Part B coverage. This exclusion, while necessary to include outpatient events, also limits generalizability to the entire U.S. ESRD population. There is also the potential for misclassification of the outcomes using claims data. We cannot address the question of whether SLE-ESRD and other ESRD patients who start hemodialysis with a permanent access in place at dialysis start have the same rates of access maturation and eventual use, because administrative data do not capture which access was used during each dialysis session. As with all observational studies, residual confounding by unmeasured factors such as differences in training and experience of regional vascular access surgeons [24] is possible. There is also the possibility of over-adjustment, given that some variables may be mediators as well as confounders and that our power was limited due to small sample sizes in some subgroups, such as SLE patients with AVGs. However, we found that, after adjustment for age, further adjustment did not change results substantially. Sensitivity of the attribution of ESRD cause to SLE on the CMS-2728 has been suggested to be low [25], although our more recent study suggested much higher sensitivity (79 %) for the capture of U.S. patients with a validated diagnosis of SLE who have progressed to ESRD [26]; however, many SLE patients may remain in the comparison group. Misclassification of access revision could have occurred by including placement of a new access in the definition, since accesses may be placed for other reasons, including aneurysm formation and steal syndrome. Data are from 2010 and may not reflect current clinical practice regarding vascular access placement and revisions generally, or specifically in SLE patients. Finally, while we performed multiple sensitivity analyses, we cannot fully address the possibility of missed events in the first 90 days of dialysis, which may differentially occur in SLE-ESRD patients.

## Conclusion

We found that SLE-ESRD patients were not more likely to have a permanent access placed in the first year of dialysis, despite a substantial gap in access placement at the start of hemodialysis [12] and despite an observed lack of association of SLE status with subsequent loss of access patency. SLE-ESRD patients are seen by multiple providers, potentially increasing opportunities for shared decision-making and coordinated care; yet, this population does not have adequate hemodialysis vascular access. Future studies should focus on provider and patient perceptions of permanent vascular access for hemodialysis in SLE-ESRD and, more generally, on the roles of nephrologists, rheumatologists, and patients in ensuring high-quality care and optimal outcomes in hemodialysis.

## Abbreviations

AVF, arteriovenous fistula; AVG, arteriovenous graft; BMI, body mass index; CMS, Centers for Medicare & Medicaid Services; ESRD, end-stage renal disease; HCPCS, healthcare common procedure coding system; SLE, systemic lupus erythematosus

## Additional file

Additional file 1: Table S1. Healthcare Common Procedure Coding System codes used to identify vascular access events within 1 year of dialysis start in U.S. patients initiating hemodialysis in 2010. (DOCX 15 kb)
