# Supplementary material for: Comparison of vascular access outcomes in patients with end-stage renal disease attributed to systemic lupus erythematosus vs. other causes: a retrospective cohort study
Source: BMC Nephrol. 2016 Jul 7;17:64. doi: 10.1186/s12882-016-0274-y (PMC4936281; doi:10.1186/s12882-016-0274-y)
Supplement: Additional file 1: Table S1. — Healthcare Common Procedure Coding System codes used to identify vascular access events within 1 year of dialysis start in U.S. patients initiating hemodialysis in 2010. (DOCX 15 kb) [file 12882_2016_274_MOESM1_ESM.docx]

**Additional file 1: Table S1.** Healthcare Common Procedure Coding System codes used to identify vascular access events within 1 year of dialysis start in U.S. patients initiating hemodialysis in 2010

| **Placement of access** | | **Revision of AVF/AVG** | |
| --- | --- | --- | --- |
| **HCPCS Code** | **Description** | **HCPCS Code** | **Description** |
| ***AVF/AVG*** | | 35475 | Transluminal balloon angioplasty, percutaneous; brachiocephalic trunk or branches, each vessel |
| 36818 | Arteriovenous anastomosis, open; by upper arm cephalic vein transposition | 35476 | Transluminal balloon angioplasty, percutaneous; venous |
| 36819 | Arteriovenous anastomosis, open; by upper arm basilic vein transposition | 36147 | Introduction of needle and/or catheter, arteriovenous shunt created for dialysis (graft/fistula); initial access with complete radiological evaluation of dialysis access, including fluoroscopy, image documentation and report (includes access of shunt, injection(s) of contrast, and all necessary imaging from the arterial anastomosis and adjacent artery through entire venous outflow including the inferior or superior vena cava) |
| 36820 | Arteriovenous anastomosis, open; by forearm vein transposition | 36148 | Additional access for therapeutic intervention |
| 36821 | Arteriovenous anastomosis, open; direct, any site (e.g., Cimino type) (separate procedure) | 36831 | Thrombectomy, open, arteriovenous fistula without revision, autogenous or nonautogenous dialysis graft (separate procedure) |
| 36825 | Creation of arteriovenous fistula by other than direct arteriovenous anastomosis (separate procedure); autogenous graft | 36832 | Revision, open, arteriovenous fistula; without thrombectomy, autogenous or nonautogenous dialysis graft (separate procedure) |
| 36830 | Creation of arteriovenous fistula by other than direct arteriovenous anastomosis (separate procedure); nonautogenous graft (e.g., biological collagen, thermoplastic graft) | 36833 | Revision, open, arteriovenous fistula; with thrombectomy, autogenous or nonautogenous dialysis graft (separate procedure) |
| ***Catheter*** | | 36838 | Distal revascularization and interval ligation (DRIL), upper extremity hemodialysis access (steal syndrome) |
| 36558 | Insertion of non-tunneled centrally inserted central venous catheter, without subcutaneous port or pump; age 5 years or older | 36870 | Thrombectomy, percutaneous, arteriovenous fistula, autogenous or nonautogenous graft (includes mechanical thrombus extraction and intra-graft thrombolysis) |
| 36565 | Insertion of tunneled centrally inserted central venous access device, requiring 2 catheters via 2 separate venous access sites; without subcutaneous port or pump (e.g., Tesio type catheter) | 37236 | Transcatheter placement of an intravascular stent (s) (except lower extremity, cervical carotid, extracranial vertebral or intrathoracic carotid, intracranial, or coronary), open or percutaneous, including radiological S&I and all angioplasty within the same vessel, when performed; initial artery |
|  |  | 37237 | Each additional artery |
|  |  | 37238 | Transcatheter placement of an intravascular stent (s)open or percutaneous, including radiological S&I and angioplasty within the same vessel, when performed; initial vein |
|  |  | 37224 | Each additional vein |
|  |  | 37239 | Revascularization, endovascular, open or percutaneous, femoral, popliteal artery(s), unilateral; with transluminal angioplasty |

AVF, arteriovenous fistula; AVG, arteriovenous graft; HCPCS, Healthcare Common Procedure Coding System.
